# The Influences of Omega-3 Polyunsaturated Fatty Acids on the Development of Skin Cancers

**DOI:** 10.3390/diagnostics11112149

**Published:** 2021-11-19

**Authors:** Yoko Minokawa, Yu Sawada, Motonobu Nakamura

**Affiliations:** Department of Dermatology, University of Occupational and Environmental Health, 1-1, Iseigaoka, Yahatanishi-Ku, Kitakyushu 807-8555, Japan; min.yo5.0628@gmail.com (Y.M.); motonaka@med.uoeh-u.ac.jp (M.N.)

**Keywords:** omega-3 PUFA, melanoma, basal cell carcinoma, lymphoma, squamous cell carcinoma

## Abstract

Dietary nutrition intake is essential for human beings and influences various physiological and pathological actions in the human body. Among various nutritional factors, dietary intake of omega-3 polyunsaturated fatty acids (PUFAs) has been shown to have various beneficial effects against inflammatory diseases. In addition to their therapeutic potency against inflammation, omega-3 PUFAs have also been shown to have anti-tumor effects via various mechanisms, such as cell arrest and apoptosis. To date, limited information is available on these effects in cutaneous malignancies. In this review, we focused on the effect of omega-3 PUFAs on skin cancers, especially malignant melanoma, basal cell carcinoma, lymphoma, and squamous cell carcinoma and discussed the detailed molecular mechanism of the omega-3 PUFA-mediated anti-tumor response. We also explored the molecular mechanisms mediated by epigenetic modifications, cell adhesion molecules, and anti-tumor immune responses.

## 1. Introduction

The skin is a representative peripheral lymphoid tissue and is the outermost organ of the human body [1,2,3]. It is assumed that the skin is one of the sites most exposed to environmental factors [4,5,6], which occasionally triggers skin oncogenesis. Malignant tumors are derived from normal host body cells and often show unfavorable behaviors, such as invasion and metastasis, leading to fatal clinical outcomes. As the incidence of skin cancers has gradually increased worldwide, clinicians should focus on the prevention of cutaneous malignancies.

Nutrition from food is essential for sustaining lives and maintaining body structures in animals. Among various nutrient factors, fatty acids are a component of the cell membrane and contribute to signal network transduction in the body. Although there are many types of fatty acids with various physiological and pathological actions [7,8,9,10], omega-3 fatty acids, found in fish oil and nuts, have beneficial effects against human diseases and inflammatory responses [11]. In addition to their therapeutic potency [12,13,14], omega-3 fatty acids may also play a role in the development of cutaneous malignancies due to their anti-inflammatory actions [15]. In this review, we have focused on the impact of omega-3 fatty acids on cutaneous malignancies and discussed the detailed molecular mechanisms in addition to recent novel findings.

## 2. Omega-3 Fatty Acids

Omega-3 polyunsaturated fatty acids (PUFAs) are found in fish oil and nuts. These fatty acids consist of ≥18 carbon chains, including a double bond of three atoms that are away from the terminal methyl group. Omega-3 PUFAs have been reported including docosahexaenoic acid (DHA) and eicosapentaenoic acid (EPA). ALA is first converted to EPA and subsequently to DHA [11]. The liver is primarily responsible for these conversions. However, conversion enzymes are extremely limited in the human body [11]. Therefore, to obtain their benefits, the direct intake of DHA and EPA from food and supplements is necessary.

The omega-3 PUFAs are essential nutrients and cannot be synthesized de novo in sufficient quantities for normal physiological function [11]. α-linolenic fatty acid is an omega-3 PUFA member and a precursor of other omega-3 PUFAs mediated by elongase and desaturase activities. In addition, α-linolenic acid is an essential fatty acid because humans do not possess the enzymes to synthesize the compound; therefore, it must be obtained from dietary sources. α-linolenic fatty acid is first metabolized by ∆6-desaturase and subsequently elongase, leading to the conversion into EPA following ∆5-desaturase activity. Further elongase and ∆4-desaturase or ∆6-desaturase activity finally produce DHA [16].

Omega-3 PUFAs have anti-inflammatory effects against inflammatory diseases, including inflammatory bowel disease, psoriasis, and rheumatoid arthritis [11,17]. The metabolites of omega-3 PUFAs, such as resolvins, also demonstrate a strong anti-inflammatory potential. E-series resolvins are derived from EPA via the acetylated cyclooxygenase-2 or cytochrome P450 pathways. In contrast, D-series resolvins and protectins are converted from DHA by 15-lipoxygenase. Their metabolites have shown strong anti-inflammatory effects in various skin disease models [12,13,14]. These metabolites have also demonstrated anti-inflammatory effects in inflammatory disease models, such as psoriasis, contact hypersensitivity, asthma, and colitis. However, limited information is available on their role in malignancies, especially skin cancers.

## 3. Influence of Omega-3 Fatty Acids on Skin Cancers

Various malignancies are influenced by omega-3 PUFAs. In this section, we detail the effects of omega-3 PUFAs on skin cancers, especially malignant melanoma, squamous cell carcinoma, basal cell carcinoma, and lymphoma.

### 3.1. Malignant Melanoma

Melanoma is a melanocyte-derived malignancy with an unfavorable clinical course without radical treatment, as it is resistant to current chemotherapies [18,19]. Although current immune checkpoint inhibitor treatment and BRAF-targeted agents have dramatically expanded the therapeutic options and improved clinical outcomes, clinical outcomes are unsatisfactory. Therefore, some additional therapeutic options for the treatment of malignant melanoma are highly desirable.

**Figure 1:**
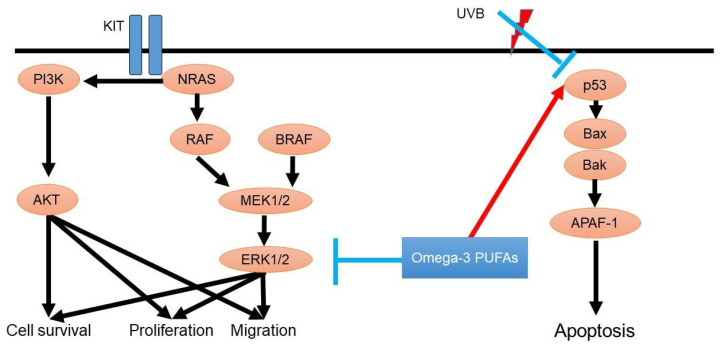
Oncogenesis signaling of malignant melanoma and possible roles of omega-3 PUFAs. BRAF plays a role in MAPK pathway activation, which regulates the development of tumor cells, such as apoptosis, cell growth, and proliferation. NRAS mutations are related to the regulation of PI3K. Ultraviolet light exposure dysregulates p53 expression, leading to the suppression of tumor cell apoptosis.

The BRAF gene is located on chromosome 7 (7q34). It encodes the BRAF protein [20] and plays a role in the mitogen-activated protein kinase (MAPK) pathway activation [21]. This activation regulates the development of tumor cells, including apoptosis, cell growth, and proliferation (Figure 1). BRAF gene mutations impair these functions. The BRAF^V600^ mutations are a representative gene mutation in melanoma and are detected in approximately 50% of patients with malignant melanoma. This gene mutation causes activation of the MAPK downstream pathway [22]. The second most frequent mutation in malignant melanoma is NRAS, which is related to the regulation of PI3K and MAPK [23]. In acral and mucosal melanoma, C-KIT plays an important role in the malignant process [24]. Currently, there are several therapeutic options for advanced malignant melanoma. For example, immunotherapy against melanoma is currently being developed, including PD-1-or CTLA-4-targeted therapy [25]. Moreover, BRAF-targeted therapy may add another strategy for improving clinical outcomes in patients with BRAF-mutated melanoma.

As the risk factor of malignant melanoma, ultraviolet light exposure is one of the representative oncogenesis factors. UV radiation induces dysregulation of p53 in melanocytes, which is known to be a tumor suppressor gene, and contributes to the suppression of tumor apoptosis [26].

Several studies have reported that a decreased risk of melanoma is related to the intake of omega-3 PUFAs [27]. In one study, the intake of EPA or DHA was associated with an approximately 80% lower risk of malignant melanoma [27]. To optimize the benefit of omega-3 PUFAs, DHA was used in combination with chemotherapy. DHA plus doxorubicin has shown greater anti-tumor efficacy than doxorubicin alone in melanoma cells [28]. In one study, when DHA–paclitaxel was administered to patients with cutaneous and mucosal metastatic melanoma, 10% of patients had partial responses and 50% of patients had stable disease [29]. In another study, the efficacy of DHA–paclitaxel was demonstrated in patients with metastatic uveal melanoma. Among patients with liver metastases, 4.5% had a partial response lasting for 5 months and 32% had stable disease [30]. Dacarbazine is currently used for melanoma treatment. In a study of 393 chemotherapy-naive patients with metastatic melanoma, DHA–paclitaxel was found to be not inferior to dacarbazine [31].

Linoleic acid, EPA, and DHA can suppress murine melanoma cell growth [32]. Intravenously injected malignant murine melanoma cells cultured with EPA have shown impaired tumor invasion and metastasis. EPA inhibits the lipoxygenase pathway, which is required for tumor metastasis [33]. COX-2 is essential for tumor invasion. TNF-α upregulates COX-2 expression in melanoma cells, which is decreased in melanoma cells co-cultured with EPA or DHA, subsequently decreasing tumor invasion [34]. DHA also decreases the expression and stability of COX-2 mRNA [35], while CXCR4 contributes to the progression of melanoma. In contrast, EPA decreases CXCR4 levels [36]. DHA inhibits cell growth and reduces invasion and migration by inhibiting matrix metalloproteinases [37]. Furthermore, algal oil, which is rich in omega-3 PUFAs, has been shown to suppress lung metastasis of B16F10 melanoma by an autophagy mechanism mediated by inactivation of the p38 MAPK and mammalian target of rapamycin (mTOR) and activation of c-Jun N-terminal kinases (JNKs), which leads to the suppression of proinflammatory cytokine production [38].

As the detailed molecular mechanism of the anti-cancer effects of omega-3 PUFAs, the suppression of debris-induced tumor growth activation has been elucidated [39]. DHA and EPA induce the release of calcium and reactive oxygen species (ROS) production, which induces the ER stress-triggered apoptosis pathway and tumor suppressor gene induction, such as P53 [40]. In one study, decreased proliferation associated with oxidative protein and DNA damage was observed in EPA- and DHA-treated melanoma cells [41].

Although the synthase for omega-3 PUFA is limited in the human body, Fat-1 can convert omega-6 to omega-3 fatty acids. In transgenic mice, this showed a reduction in tumor growth. In vitro experiments have shown that the addition of EPA or PGE_3_ inhibited the growth of melanoma cells [42]. Treatment with omega-3-rich fish oil has also been shown to reduce melanoma development and leukotriene B4 (LTB4) production [43]. DHA inhibits cell growth in addition to the expression of cyclins and cyclin-dependent kinase inhibitors and the expression of pRb. DHA treatment results in an increase in hypophosphorylated Rb [44]. DHA also suppresses human melanoma cell growth in vivo. In animals, treatment with an omega-3 PUFA-enriched diet has demonstrated effectiveness in reducing tumors (69% smaller in weight of the tumor and 76% smaller in volume) compared to treatment with an omega-6 fatty acid-enriched diet [45].

Omega-3 PUFAs enhance the efficacy of anti-cancer agents, such as cisplatin (CDDP). EPA- and DHA-treated melanoma cells are sensitive to CDDP-induced suppression of tumor growth and invasion. Omega-3 PUFAs also drive the expression of ERCC1, DUSP6, and p-ERK, which activates ERCC1 and thereby improves the anti-tumor action of CDDP against melanoma cells [46].

Connexin 43 is related to chemosensitivity. It is suppressed in the tumor microenvironment. EPA improves chemosensitivity in murine melanoma by inducing connexin 43 expression, which is regulated by MAPK pathways [47]. In addition, DHA enhances the sensitivity of various tumor cells to ROS-inducing anti-cancer agents [48].

In contrast, another study reported an opposite effect. Fish oil EPA-28 enriched in EPA and DHA increases tumor growth and metastasis of subcutaneously injected B16 melanoma cells [49]. EPA-28 suppresses CD4^+^ T cell infiltration around the tumor in addition to the cytolytic activity of T cells and macrophages. However, the inflammatory tumor microenvironment drives tumor development. Debris derived from tumor cells in chemotherapy stimulate primary tumor growth, which is inhibited by anti-inflammatory agents that are known to have anti-tumor effects. For example, resolvin D1 (RvD1), RvD2, or RvE1 inhibits debris-stimulated cancer progression by enhancing the clearance of debris through macrophage phagocytosis in various types of tumors and suppresses inflammatory cytokines/chemokines by tumor cell debris-triggered human macrophages [50]. Overall, omega-3 PUFAs are expected to suppress melanoma development. However, omega-3 PUFAs may be disadvantageous in certain conditions in which anti-tumor immune responses to melanoma are expected.

### 3.2. Basal Cell Carcinoma and Omega-3 Fatty Acids

Basal cell carcinoma is a malignancy derived from epidermal basal keratinocytes and is commonly observed in sun-exposed sites. The clinical behavior is basically slow progressive local site invasion. Distant metastasis is rarely observed. Although surgical treatment is the gold standard for limited localized basal cell carcinoma, there is currently no therapeutic option for distant metastasis of invaded and unresectable basal cell carcinoma. Therefore, prevention is important.

**Figure 2:**
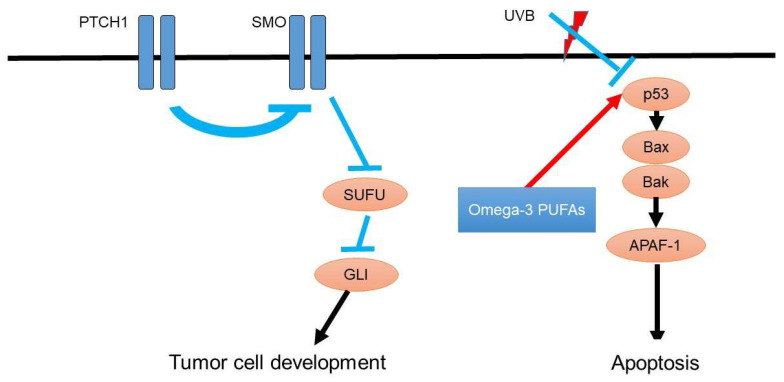
Oncogenesis signaling of basal cell carcinoma and possible roles of omega-3 PUFAs. As the detailed oncogenesis of basal cell carcinoma, hedgehog pathway gene mutation activates patched homolog and smoothened homolog, which are responsible for the development of basal cell carcinoma. P53 is suppressed by ultraviolet light exposure, leading to the suppression of apoptosis of basal cell carcinoma.

As the detailed oncogenesis of basal cell carcinoma, the hedgehog pathway gene mutation activates the patched homolog and smoothened homolog (Figure 2). Consistently, inhibition of this hedgehog signaling pathway suppresses the metastasis of basal cell carcinoma [51]. In addition to the hedgehog pathway, p53 mutations are also involved in the development of basal cell carcinoma [52]. For instance, ultraviolet light exposure induces p53 mutations and leads to tumorigenesis of basal cell carcinoma [53].

Ultraviolet light exposure also enhances the risk of basal cell carcinoma and downregulates p53 expression in the basal layer of the epidermal keratinocyte, leading to the contribution of the development of basal cell carcinoma [54].

A limited number of studies have focused on the efficacy of omega-3 PUFAs. One epidemiological study suggests that there is no clear evidence of an association between the intake of omega-3 fatty acids and the risk of basal cell carcinoma. Intake of linolenic acid was also negatively related to the risk of basal cell carcinoma [55]. Conversely, a Mendelian randomization study using PUFA level genome-wide association studies showed that alpha-linolenic acid and linoleic acid are associated with a lower risk of basal cell carcinoma [56].

### 3.3. ATLL and Omega-3 Fatty Acids

Adult T-cell leukemia/lymphoma is a malignancy related to human T-cell lymphotropic virus type I (HTLV-1)-infected mature CD4^+^ T cells [57,58]. Adult T-cell leukemia/lymphoma is classified into four clinical groups according to Shimoyama’s classification based on the severity, the number of abnormal lymphocytes, and organ involvement [58]. Skin lesions are observed in approximately 50% of adult T-cell leukemia/lymphoma patients. Assessment of skin lesions helps predict prognosis [59,60,61]. Although aggressive types—the acute and lymphoma types—of adult T-cell leukemia/lymphoma tend to have an unfavorable clinical course [62,63,64], the chronic and smoldering types are generally indolent and may be managed with “watchful waiting” [65].

A limited number of studies have focused on the therapeutic potential of ATLL. In one study, the combination of DHA and arsenic trioxide, interferon-alpha, and emodin showed a potent synergistic effect on cell cycle arrest and cell death in HTLV-1 infected cells [66]. This finding suggests that DHA is a possible candidate for the treatment of ATLL to obtain additional therapeutic effects.

### 3.4. Diffuse Large B-Cell Lymphoma and Omega-3 Fatty Acids

Diffuse large B-cell lymphoma is a malignancy of the B cells. It is a common type of non-Hodgkin cutaneous lymphoma [67]. Diffuse large B-cell lymphoma is commonly observed in the extremities and clinically manifests as solid nodules or tumors in the skin.

Dietary products with higher fat contents are positively associated with the risk of non-Hodgkin lymphoma, whereas a high intake of omega-3 fatty acids and seafood is inversely associated with the risk [68]. A low intake of omega-3 fatty acids is associated with unfavorable survival in patients with diffuse large B-cell lymphoma [69].

### 3.5. Cutaneous Squamous Cell Carcinoma (SCC)

Cutaneous SCC is a keratinocyte-derived cutaneous malignancy of the skin. Due to its increasing incidence worldwide, cutaneous SCC is a hot topic among clinicians [70,71]. Although SCC limited to the skin can be treated with surgical resection, cutaneous SCC with distant metastasis exhibit an unfavorable clinical course due to a limited number of effective therapeutic options [72,73].

**Figure 3:**
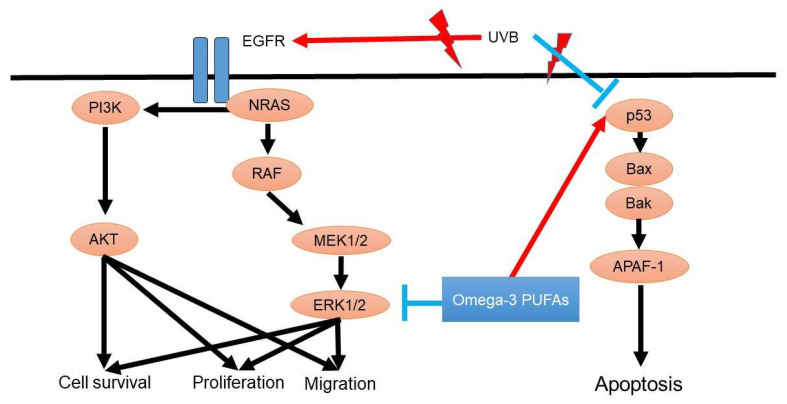
Oncogenesis signaling of squamous cell carcinoma and possible roles of omega-3 PUFAs. P53 gene mutation is a crucial step in the development of cutaneous squamous cell carcinoma. P53 is responsible for cell apoptosis, DNA repair, and cell cycle arrest in squamous cell carcinoma. In addition, EGFR-mediated PI3K/AKT signaling and NRAS/RAF/MEK/ERK signaling also contribute to the development of squamous cell carcinoma.

The p53 tumor suppressor gene is associated with oncogenesis in cutaneous SCC. P53 is crucial for the development of cutaneous SCC because p53 is responsible for cell apoptosis, DNA repair, and cell cycle arrest. In addition, EGFR-mediated PI3K/AKT signaling and NRAS/RAF/MEK/ERK signaling also contribute to the development of SCC (Figure 3).

PUFAs show an inhibitory effect on prostaglandin production by squamous carcinoma cells [74], which plays a role in the development of SCC. Extracellular signal-regulated kinase promotes cell apoptosis after omega-3 PUFA treatment. DHA promotes ROS production and activates c-Jun N-terminal kinase, leading to the enhancement of toxicity to normal keratinocytes [75]. RvD2 significantly reduces tumor size and cancer-derived cytokines/chemokines (TNF-α, IL-6, CXCL10, and MCP-1) [76]. EPA inhibits cell division [77] by releasing Ca^2+^ from intracellular stores, leading to the activation of protein kinase R, which causes cell cycle arrest in G1. Oral administration of EPA reduces tumor size by decreasing the expression of cyclin D1 in tumors. Omega-3 PUFAs inhibit tumor growth by combining with ionizing radiation [78]. Omega-3 PUFAs suppress angiogenesis, tumor proliferation, and cyclooxygenase-2 production.

Cancer-associated weight loss is an important issue in the treatment of malignancies. EPA has been shown to be potent in suppressing this response [79]. Seventy percent of the subjects who consumed EPA-containing food maintained or gained body weight. An EPA-containing nutritional supplement may also increase or maintain the body weight of patients with SCC-associated body weight loss.

Another randomized, single-blind, placebo-controlled clinical trial was conducted in patients with SCC to confirm the efficacy of EPA during treatment. In this study, EPA-supplemented diets decreased inflammatory cytokines and maintained body weight [80].

Furthermore, EPA may suppress the migration of SCC cells and reduce migration velocity and directionality in a dose-dependent manner [81].

## 4. Summary of the Effect of Omega-3 Fatty Acids against Each Skin Cancer

The detailed direct effects of omega-3 fatty acids against skin cancers are summarized and shown in Table 1. Epidemiological analyses have shown the beneficial potency of omega-3 PUFAs in melanoma [27], basal cell carcinoma [56], and diffuse large B cell lymphoma [69]. In addition, omega-3 PUFAs suppress tumor growth in melanoma [32,37,41,42,44,45] and squamous cell carcinoma [77], inhibiting migration and invasion in melanoma [33,34,37] and squamous cell carcinoma [81]. Omega-3 PUFAs also enhance the sensitivity to chemotherapy in melanoma [28,29,30,31,46,47,48] and radiosensitivity in squamous cell carcinoma [78]. Omega-3 PUFAs metabolites RvD1, RvD2, and RvE1 suppress debris-stimulated cancer progression in melanoma [50], and RvD2 reduces tumor size and cancer-derived cytokines/chemokines [76].

## 5. Epigenetic Modification by Omega-3 Fatty Acids

Normally, DNA is considered a single-line construction. However, DNA does not exist as a single line. DNA in the body is entwined with a DNA-binding protein called histone. Furthermore, it is packed into histones. When the bond between DNA and histone is loosened, gene transduction begins to read the DNA information. In vivo, the histone or DNA chemical modification-mediated epigenetic mechanism is a powerful regulatory mechanism of gene transduction [82,83]. These epigenetic modifications are involved in various human diseases [82,83,84]. Recent studies have reported that bacteria-derived short-chain fatty acids can modify histone acetylation by suppressing HDACs, creating open chromatin sites and inducing gene transduction [83].

A recent study demonstrated that omega-3 fatty acids may have a role in epigenetic modification, which in turn, has significant multiple anti-tumor roles [85]. There is an interaction between the anti-tumor role of omega-3 PUFAs and the DNA demethylation pathway leading to the anti-cancer mechanism of omega-3 PUFAs mediated by altering the patterns of ten-eleven translocation (TET) 1 expression.

TET1 is also involved in cancers such as basal cell carcinoma [86], SCC [87], melanoma [88], and lymphomas [89]. UV radiation is involved in the pathogenesis of tumor development related to TET1. In the normal skin and cutaneous cell lines, UVB exposure induces the upregulation of DNMT1 and downregulates TETs [87]. In mouse models, silencing of DNMT1 and overexpression of TET1 and TET2 can lead to an increase in ID4 expression [87], which in turn, reduces cell proliferation, migration, and invasion, increases apoptosis in cutaneous SCC cell lines, and reduces tumorigenesis [87]. TET1 is also downregulated on NF-κB activation through the binding of p65 to its consensus sequence in the TET1 promoter [88].

## 6. P53 and Omega-3 PUFAs

p53 is a classic tumor suppressor gene that is responsible for the induction of cell apoptosis and cell cycle arrest. p53 is involved in tumorigenesis and the development of tumors. There are various studies regarding the efficacy of omega-3 PUFAs on p53 suppression, such as in colorectal adenocarcinoma [90,91]. Although the effect of omega-3 PUFAs on p53 expression in skin cancers is unclear, further investigation to clarify the actual impact of omega-3 PUFAs should be considered.

## 7. ERK Inhibition and Omega-3 PUFAs

The MEK–ERK–BRAF pathway has been highlighted by clinicians because the efficacy of BRAF and/or BRAF-related pathway inhibition dramatically improves anti-tumor effects. RAS is a non-BRAF oncogene activated in malignant melanoma and enhances tumor functions, such as cell growth and apoptosis. RAS promotes the downstream pathways of MEK/ERK, MAPK, and PI3K/AKT signaling. However, BRAF mutation in melanoma, especially BRAFV600 mutations, activates the downstream pathway of MAPK and positively drives the development of malignant melanoma.

Several studies have reported the anti-tumor effect of omega-3 PUFAs against malignancies, but not in skin cancers. Omega-3 PUFAs have been shown to suppress tumor growth mediated by ERK suppression in retinoblastoma [92], leukemia cells [93], breast cancer [94], and colon cancer [95]. The effect of omega-3 PUFAs on melanoma cells is unclear. Therefore, the detailed molecular mechanism of the anti-tumor effect of omega-3 PUFAs on melanoma should be investigated.

## 8. Adhesion Molecule and Omega-3 PUFAs

Cell–cell adhesion is important in maintaining the physiology of cell function. Its abnormality changes the structure and enhances the dissemination and metastatic spread of tumor cells. A component of the adhesion molecule is E-cadherin, and cell adhesion molecule 1 (CADM1) plays a role in cell–cell adhesion, and low expression of these adhesion molecules is related to lower survival in solid tumors. Cell adhesion is essential to the tumor environment. Reduced expression of the cell adhesion molecules plays a pivotal role in the metastatic ability of the tumor [96,97,98]. Although there is no clear evidence demonstrating that omega-3 PUFAs modify the expression of these cell adhesion molecules in skin cancers, EPA increases the expression of E-cadherin in colon cancer and breast cancer [99].

In addition to cell–cell adhesion, vascular cell adhesion molecules also play an important role in the distant metastasis of the tumor. High expression of vascular cell adhesion molecules becomes a scaffold for cell invasion into the blood vessels and enhances the migration of tumor cells. There is no report regarding the effect of omega-3 PUFA on the vascular cell adhesion molecules focused on the skin cancers-bearing host body. However, vascular cell adhesion molecule 1 expression on ACL-15 cells is downregulated by EPA–ethyl ester treatment [100], suggesting that a decreased ability of tumor cell adhesion to the capillary bed is expected during the treatment of omega-3 PUFA in patients with skin cancers.

## 9. Anti-Tumor Immunity and Omega-3 PUFAs

Immune checkpoint inhibitor treatment currently improves clinical outcomes in patients with unresectable skin cancers with distant metastasis [101,102,103]. Although there are several adverse reactions [104], some additive therapeutic potential is desired with the combination of immune checkpoint inhibitor treatment [105]. Although omega-3 PUFAs show an anti-tumor effect in skin cancers, it may be disadvantageous to use omega-3 PUFAs in combination therapy with immune checkpoint inhibitor treatment.

PUFAs regulate T-cell activation [106]. After culture with DHA, DCs expressed higher levels of CD40, CD80, CD83, CD86, and PDL-1. However, fewer T cells co-cultured with these DCs proliferated, while an increased Treg phenotype was observed in co-cultures with DHA-primed DCs.

## 10. Conclusions

Omega-3 PUFAs influence the proliferation and metastasis of various skin cancers Basically, omega-3 PUFAs show beneficial potency against skin cancers. Therefore, the intake of omega-3 PUFAs may be recommended. However, omega-3 PUFAs suppress the immune reaction, mediated mainly by Tregs, possibly leading to the impairment of anti-tumor immune reactions. Based on this mechanism, a combination of omega-3 PUFAs and an immune checkpoint inhibitor may not be recommended for use in the treatment of malignancies. However, specialized lipid mediators, such as resolvins and protectins, potently suppress tumor-derived dust-induced inflammatory responses in the induction of tumor development. Thus, omega-3 PUFAs may positively drive the anti-tumor effect in skin cancers.

## Figures and Tables

**Table 1:** Summary of detailed action of omega-3 PUFAs against skin cancers.

Skin Cancers	Detailed Action
Melanoma	Reduce the risk of melanoma by DHA/EPA intake [27] Enhance the sensitivity to chemotherapy by EPA and DHA [28,29,30,31,46,47,48]. Suppress tumor growth by linoleic acid, EPA, DHA [32,37,41,42,44,45]. Suppress tumor metastasis by EPA [33,37] Suppress tumor invasion by EPA and DHA [34,37] Enhance ROS production and P53 induction by DHA and EPA [40]. Suppress debris-stimulated cancer progression by RvD1, RvD2, or RvE1 [50]
BCC	Reduce the risk of BCC by α-linolenic acid and linoleic [56].
ATLL	Synergistic effect on cell cycle arrest and cell death by the combination of DHA with chemotherapy [66].
DLBCL	Low intake of omega-3 fatty acids is associated with unfavorable survival [69].
SCC	Enhance the toxicity by DHA [75]. Reduce tumor size and cancer-derived cytokines/chemokines by RvD2 [76]. Suppress cell proliferation by EPA [77] Suppress tumor growth by the combination of Omega-3 PUFAs with ionizing radiation [78]. Suppress migration by EPA [81].

## Data Availability

Not applicable.
